# Danshen (*Salvia miltiorrhiza*) extract in spinal cord injury (SCI): a systematic review and meta-analysis

**DOI:** 10.3389/fphar.2026.1754584

**Published:** 2026-04-20

**Authors:** Yuanna Zhang, Dongping Wan, Rui Tang, Rui Wang, Haodong Wu, Feilong Li, Xiang Ji, Xi Gao, Shihang Cao

**Affiliations:** 1 Honghui Hospital, Xi’an Jiaotong University, Xi’an, Shaanxi Province, China; 2 The First Clinical Medical College, Guangxi University of Chinese Medicine, Nanning, Guangxi Zhuang Autonomous Region, China; 3 The Clinical Medical College, Chengdu University of Traditional Chinese Medicine, Chengdu, Sichuan Province, China; 4 Department of Orthopedics and Traumatology, The Affiliated Traditional Chinese Medicine Hospital, Southwest Medical University, Luzhou, Sichuan Province, China

**Keywords:** Danshen, Danshen extract, meta-analysis, *Salvia miltiorrhiza*, spinal cord injury

## Abstract

**Purpose:**

This study aims to systematically assess the effectiveness of Danshen (*Salvia miltiorrhiza*) extract in animal models of spinal cord injury (SCI) and provide high-quality evidence to support the translation from preclinical research to clinical practice.

**Methods:**

A systematic review and meta-analysis of 35 randomized controlled trials involving animal models of SCI were conducted. Data were extracted regarding the effects of Danshen extract on motor function (measured by the BBB score), inflammation, oxidative stress, apoptosis, and edema. Subgroup analyses based on model type, drug dosage, and compound type were also performed.

**Results:**

Danshen extract significantly improved motor function at multiple time points (3, 7, 14, and 21 days post-injury). The BBB scores were higher in the treatment group across all time points (SMD = 4.53 at 3 days, p < 0.001). Additionally, Danshen reduced inflammatory markers (TNF-α, IL-1β), oxidative stress (MDA), and apoptotic markers (Caspase-3), while increasing antioxidant activity (SOD). It also significantly reduced spinal cord edema, as indicated by decreased water content in the injury areas (SMD = −3.88, p < 0.00001). Subgroup analysis showed the most significant improvements in contusion and ischemic models, with higher doses (>20 mg/kg) and water-soluble phenolic acids providing the best outcomes.

**Conclusion:**

Danshen extract exhibits significant potential in treating SCI through its diverse mechanisms, such as reducing inflammation, combating oxidative stress, preventing cell death, and alleviating edema. However, further research is needed to refine treatment protocols and establish its clinical relevance.

**Systematic Review Registration:**

https://www.crd.york.ac.uk/PROSPERO/recorddashboard.

## 1 Introduction

Spinal cord injury (SCI) is a catastrophic disease of the central nervous system, characterized by primary mechanical damage followed by a cascade of secondary injuries, which constitute its core pathological processes. It leads to persistent neuronal cell death, axonal degeneration, and scarring, ultimately resulting in long-term motor and sensory deficits as well as significant socio-economic burdens (Hachem and Fehlings, 2021; Ahuja et al., 2017; Alizadeh et al., 2019). SCI is a major cause of terminal neurofunctional loss and disability (Ding et al., 2022; Truong et al., 2025). Falls and road traffic accidents are the two leading causes globally (GBD Spinal Cord Injuries Collaborators, 2023). The secondary injury evolves in a time-dependent manner, involving oxidative stress, inflammation amplification, excitotoxicity, mitochondrial dysfunction, blood-spinal cord barrier disruption, and microcirculatory disturbances, providing potential windows for neuroprotective and repair interventions (Cavalcanti et al., 2025; Hejrati et al., 2025; Kandemir et al., 2025; Oyinbo, 2011). Additionally, SCI induces multi-system effects through neuroimmune and vascular pathways, contributing to increased risks of neuropathic pain, respiratory dysfunction, cardiovascular complications, and neurogenic bladder (Luo et al., 2024; Hirota et al., 2022; Xu et al., 2025c; Aude et al., 2025).

In areas with a high occurrence of traumatic incidents like traffic collisions and sports-related injuries, the rate of SCI continues to be significantly elevated (Liu et al., 2025). Although the global overall burden (incidence, prevalence, and disability-adjusted life years) shows a downward trend, significant regional and population differences exist, with the burden remaining particularly prominent in males and the elderly (Lu et al., 2024). With improvements in pre-hospital care, acute-phase treatment, and nursing techniques, the mortality rate of SCI patients has decreased from 4.42% to 0.44% (Huang et al., 2019). However, current treatments still struggle to effectively restore neurological function, constrained by disease heterogeneity, limited therapeutic targets, and safety concerns (Hu C-W. et al., 2025). The benefit-risk ratio of corticosteroids (e.g., methylprednisolone) remains controversial, and their routine use is no longer recommended, prompting research to shift towards multi-target, low-toxicity alternative strategies (Geisler et al., 2023).

In recent years, natural substances have gained significant interest due to their ability to act on multiple biological pathways and targets, making them a promising area in SCI treatment (She et al., 2025). Danshen extract, obtained from *Salvia miltiorrhiza*, is a traditional herbal remedy recognized for its properties in reducing inflammation, combating oxidative stress, preventing cell death, and offering neuroprotective effects (Su et al., 2015; Jung et al., 2020). Due to its distinct chemical composition and broad range of therapeutic effects, Danshen extract is commonly utilized in the treatment of cardiovascular conditions, liver diseases, and neurological disorders (Orgah et al., 2020; Qiu et al., 2008; Xia et al., 2025). Danshen extract is rich in bioactive compounds, such as tanshinone IIA and salvianolic acid B, which exhibit a broad spectrum of pharmacological effects, including reducing inflammation, protecting against oxidative damage, preventing cell death, maintaining blood-brain/spinal cord barrier integrity, and improving microcirculation in various neurological injury models (Hu W. et al., 2025; Shao et al., 2025; Zhang et al., 2022; Xu M. et al., 2025). These benefits may arise from the modulation of crucial pathways, including NF-κB, Nrf2/HO-1, caspase-dependent apoptosis, and tight junction protein expression, all of which contribute to neuroprotection (Jung et al., 2020; Yin et al., 2012; Xiao et al., 2020). However, existing evidence is scattered across different species and injury paradigms (e.g., contusion, compression, transection), formulations (e.g., whole extracts, injections, monomers), administration regimens, outcome measures (behavioral, histological, and molecular-biochemical), and follow-up time points, leading to high heterogeneity. This limits the generalizability and clinical translation of the results.

Danshen extract is considered a promising candidate for the treatment of SCI (Zhang et al., 2019; Zhang et al., 2018). While previous studies have shown that herbal medicines significantly improve sensory function (ASIS-SIS), motor function (ASIS-MS), daily activities (BI), and pain levels (VAS) in SCI patients (Jiang et al., 2025), there is a lack of systematic reviews and meta-analyses specifically on the use of Danshen extract for the treatment of SCI. However, systematic evidence regarding its preclinical studies remains limited. Therefore, it is urgent to follow methodological standards for experimental animal research and conduct a systematic review and meta-analysis of SCI animal models. This will enable the quantitative assessment of the efficacy and robustness of Danshen extract, explore potential effect-modifying factors (such as model type, formulation/dose, timing of administration, follow-up duration, etc.), and systematically evaluate risk of bias and publication bias. Such efforts will provide a higher-quality evidence base for subsequent mechanistic studies and clinical trial design.

## 2 Materials and methods

This meta-analysis was performed in accordance with the PRISMA guidelines (Liberati et al., 2009) and has been registered with PROSPERO (CRD420251239662).

### 2.1 Literature search

A thorough search was conducted across both English-language databases (including EMBASE, PubMed, Web of Science, and the Scopus) and Chinese-language databases (including CNKI, Wanfang, VIP, and SinoMed). The search focused on studies investigating the impact of Danshen extract on spinal cord injury (SCI) and related outcomes in animal models, covering the period from the inception of the databases to October 2025. Keywords like ‘Danshen extract,’ ‘spinal cord injury,’ ‘preclinical study,’ and ‘animal’ were used to ensure comprehensive retrieval of relevant studies.

### 2.2 Inclusion and exclusion criteria

Eligible studies were *in vivo* experiments employing rodent models of traumatic SCI—including contusion, compression, transection, or clip-compression—in which the intervention was Dan Shen or Dan Shen Extract administered as monotherapy at a specified dose, route, and schedule; The control group was given either an equal volume of saline/vehicle or no treatment at all; and the original report provided at least one of the following prespecified outcomes: Pro-inflammatory cytokines (such as IL-1β and TNF-α), NF-κB activation markers (e.g., p65 nuclear translocation or pathway readouts), apoptotic markers (Bcl-2, caspase-9, caspase-3), and Akt protein/phosphorylation levels (e.g., p-Akt/total Akt), locomotor function (Basso–Beattie–Bresnahan [BBB] score), oxidative stress indices (MDA, SOD), TUNEL-positive cell counts, or spinal cord water content.

Initially, we reviewed the titles and abstracts of all retrieved records. Studies were excluded based on the following criteria: (1) non-original research or studies with incomplete data; (2) clinical trials, *in vitro* experiments, retrospective studies, case reports, or study protocols; (3) interventions that did not involve Danshen extract or lacked clear information on dosage and timing; (4) studies in which the SCI group received additional treatments. The authors then evaluated the full-text articles and excluded those that did not provide relevant outcome measures.

### 2.3 Data extraction

Two researchers independently reviewed and cross-checked all studies meeting the data extraction criteria. The following information was extracted from each study: the first author’s name and publication year; details of the animal model (sex, weight, sample size, and type); methods for disease modeling, including anesthetic use; treatment details for both the experimental and control groups, such as dosage, administration frequency or cycles, route, carrier name, and volume; reported outcome measures; and potential mechanisms by which Danshen extract exerts its anti-SCI effects. To evaluate the dose-response relationship, studies examining multiple doses of Danshen extract were considered, with additional analysis performed on data from the highest-dose group. Efforts were made to contact the original authors for graphical data, and for data that could not be obtained, measurement software was used for calculations.

### 2.4 Quality assessment

The risk of bias was assessed independently using the SYCLE tool, covering six domains: selection, performance, detection, attrition, reporting, and other biases. Studies meeting the criteria were considered low risk, those failing to meet the criteria were high risk, and studies with unclear bias descriptions were labeled as unclear. Disagreements were resolved through discussion.

### 2.5 Statistical analysis

Continuous variable data, including means and standard errors, were recorded in Microsoft Excel. The data were analyzed and visualized using Review Manager 5.3.0 and Stata 16.0, with statistical significance set at p < 0.05 when appropriate. The standardized mean difference (SMD) and 95% confidence intervals (95% CI) were used to quantify the results. Heterogeneity was evaluated using the I^2^ index, where studies with I^2^ < 50% were analyzed with a fixed-effect model, and those with I^2^ > 50% were analyzed using a random-effects model due to high heterogeneity. Subgroup and sensitivity analyses, based on factors such as animal species and treatment timing, were performed to confirm the reliability of the findings. Publication bias was assessed through Egger’s test, where a probability value (Prob) > 0.05 indicated no bias. Additionally, the mechanisms identified in preclinical studies were summarized.

## 3 Results

### 3.1 Retrieve results

**FIGURE 1:**
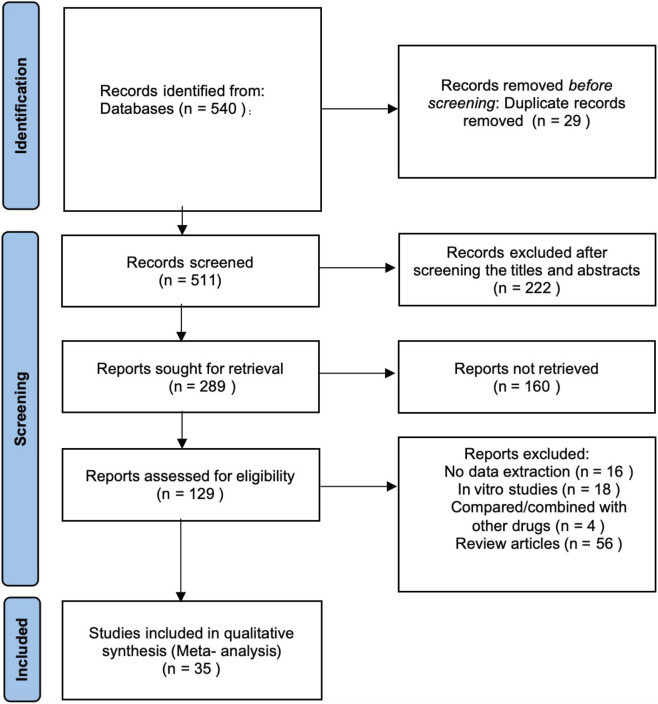
Quality of the included studies.

A total of 540 articles were identified through the initial search. Duplicates were excluded, as well as those articles whose titles and abstracts did not meet the inclusion criteria. The remaining 129 articles underwent full-text review. Out of the total, 94 articles were excluded for reasons such as being review articles, not involving rodent experiments, or lacking valid data. Ultimately, 35 randomized controlled trials were included in the meta-analysis (Figure 1).

### 3.2 Characteristics of the study

This meta-analysis included 35 (Bi et al., 2008; Cai et al., 2014; Chen et al., 2012a; Chen et al., 2012b; Chen et al., 2011; Fan et al., 2013; Fu et al., 2014; Jiang et al., 2023; Li et al., 2015; Liu et al., 2016; Liu et al., 2019; Luo et al., 2022; Nie et al., 2018; Rao et al., 2024; Sun et al., 2019; Wang et al., 2015; Wang et al., 2025; Wang et al., 2019; Wei et al., 2019; Xu et al., 2025a; Xu et al., 2025b; Xun et al., 2014; Yang et al., 2016b; Yang et al., 2017; Yao, et al., 2017; Ye et al., 2011; Yin, X. et al., 2012; Yong et al., 2016a; Yu and Qian, 2020; Yu et al., 2019; Zhang et al., 2012; Zhang et al., 2019; Zhong et al., 2025; Zhou et al., 2020; Zhu et al., 2016) animal studies evaluating Dan Shen extract for SCI, published between 2008 and 2025. Injury models comprised Allen’s weight‐drop (n=12), NYU impactor (n=5), Zivin ischemia (n=5), weight-drop impact (n=4), compressive SCI (n=2), complete transection (n=2), and single studies using aortic occlusion, contusion, screw compression, arterial clamp compression/occlusion, and aminectomy-induced injury. Thoracic SCI predominated (27/35), followed by lumbar (4/35), cervical (2/35), mixed thoraco-lumbar (1/35), with 2 unreported levels. Species were mainly Sprague–Dawley rats (n=29), with rabbits (n=4), mice (n=2), and Wistar rats (n=1); sex was male (17 studies), female (13), both (3), and unreported (3). Regarding active constituents, tanshinones were most common (20 studies; chiefly Tanshinone IIA, plus dihydrotanshinone I), followed by salvianolic acids (10 studies; mainly Sal B/total salvianolic acid), crude/extracts of S. miltiorrhiza (5), and danshensu (1). Routes of administration were predominantly intraperitoneal (26/35), with intravenous (5/35), oral gavage (3/35), subarachnoid injection (1/35), and 1 unreported. Study durations ranged from 12 hours to 8 weeks. Table 1 outlines detailed study characteristics.

**Table 1:** Characteristics of the included studies.

First author	Induction of sCI	Spinal cord	Species	Gender	Effective substance	Sample size		Intervention		Methods of administration	Duration of study
						IG	CG	IG	CG		
Bi et al. (2008)	Allen's weight-drop	TSC	SD	Female	Sal B	6	6	8 mg/kg	PBS	IP	4 wks
Chen et al. (2011)	Compressive SCI	TSC	Wistar rats	Male	Danshensu	18	18	5 mg/kg	NS	IP	10 d
Ye et al. (2011)	Allen's weight-drop	TSC	SD	Female	Sal B	30	30	20 mg/kg	PBS	IP	2 wks
Yin et al. (2012)	Allen's weight-drop	TSC	SD	Male	Tanshinone IIA	12	12	20 mg/kg	NS	IP	10 d
Chen et al. (2012a)	Zivin	——	Rabbit	Male	Tanshinone IIA	6	6	50mg/kg	NS	IP	12 h
Zhang et al. (2012)	Zivin	LSC	SD	Male/Female	Danshen extract	35	35	0.92 mg/kg	NS	IP	12 h
Zhang et al. (2012)	Zivin	LSC	SD	Male/Female	Tanshinone IIA	35	35	3.56mg/kg	NS	IP	12 h
Chen et al. (2012b)	Zivin	——	Rabbit	Male	Tanshinone IIA	6	6	50mg/kg	NS	IP	12 h
Fan et al. (2013)	Compressive SCI	TSC	SD	Male	Sal B	30	30	10mg/kg	NS	IP	3 wks
Fu et al. (2014)	Aortic occlusion	LSC	SD	Male	SalB	48	48	50mg/kg	NS	IV	2 wks
Xun et al. (2014)	Contusion method	TSC	SD	Male	Sal B	10	10	10 mg/kg	PBS	IP	4 wks
Cai et al. (2019)	Screw compression method	CSC	Rabbit	——	Tanshinone IIA	12	12	3 mg/kg	NS	——	2 wks
Li et al. (2015)	Zivin	LSC	SD	male	Total Salvianolic Acid	6	6	25 mg/kg	NS	IP	48 h
Wang et al., (2025)	Allen's weight-drop	TSC	SD	male	Salvia miltiorrhiza	15	15	30 mg/kg	NS	IP	3 wks
Liu et al. (2016)	Allen's weight-drop	TSC	SD	male	Salvia miltiorrhiza aqueous root extract	10	10	10 µM	NS	IP	11 d
Zhu et al. (2016)	Allen's weight-drop	TSC	SD	Female	Sal B	10	10	20 mg/kg	NS	IP	8 wks
Yang et al. (2016a)	Allen's weight-drop	TSC	SD	Female	Tanshinone ⅡA	10	10	20 mg/kg	NS	IV	8 wks
Yang et al. (2016b)	NYU spinal cord impactor	TSC	SD	Female	Tanshinone ⅡA	11	11	30 mg / kg	NS	IV	4 wks
Yang et al. (2017)	NYU spinal cord impactor	TSC	SD	Female	Tanshinone ⅡA	20	20	20 mg/kg	NS	IP	4 wks
Yao et al. (2017)	Allen's weight-drop	TSC	SD	male	Tanshinone ⅡA	12	12	30 mg/kg	NS	IP	1 wks
Nie et al. (2018)	Allen's weight-drop	TSC	SD	Male/Female	Tanshinone ⅡA	30	30	——	NS	IP	2 wks
Liu et al., 2019	Arterial clamp compression	TSC	SD	male	Tanshinone ⅡA	15	15	30 mg/kg	NS	IP	3 d
Sun et al. (2019)	Allen's weight-drop	TSC	SD	Female	Sal B	10	10	10 mg/kg	NS	IP	4 wks
Wang et al. (2019)	Weight-drop impact method	TSC	FVB mice	Male	Sal B	6	6	30 mg/kg	NS	Gavage	5 wks
Yu et al. (2019)	Weight-drop impact method	T-LSC	Rabbit	——	Danshen extract	16	16	0.3 ml/kg	NS	Subarachnoid injection	3 d
Zhang et al. (2019)	Weight-drop impact method	TSC	SD	Female	Danshen extract	12	12	12.5 g/kg	——	Gavage	4 wks
Liu et al. (2019)	Arterial clamp occlusion	TSC	SD	Male	Tanshinone ⅡA	6	6	30 mg/kg	NS	IP	3 d
Yu and Qian (2020)	Aminectomy-induced injury	TSC	SD	——	Dihydrotanshinone Ⅰ	8	8	4 mg/kg	NS	Gavage	4 wks
Zhou et al. (2020)	Weight-drop impact method	TSC	SD	male	Sal B	8	8	20 mg/kg	PBS	IP	8 wks
Luo et al. (2022)	Allen's weight-drop	TSC	C57	male	Tanshinone ⅡA	5	5	20 mg/kg	NS	IP	3 wks
Jiang et al. (2023)	Complete Transection Model	TSC	SD	Female	Tanshinone ⅡA	12	12	20 mg/kg	NS	IP	2 wks
Rao et al. (2024)	Complete Transection Model	TSC	SD	Female	Tanshinone ⅡA	18	18	20 mg/kg	NS	IP	8 wks
Xu et al. (2025a)	NYU spinal cord impactor	TSC	SD	female	Tanshinone ⅡA	6	6	20 mg/kg	NS	IP	7 wks
Xu et al. (2025b)	NYU spinal cord impactor	CSC	SD	female	Tanshinone IIA	15	15	20 mg/kg	NS	IV	8 wks
Zhong et al. (2025)	NYU spinal cord impactor	TSC	SD	Female	Tanshinone IIA	36	36	20 mg/kg	NS	IP	8 wks
Wang et al. (2025)	Allen's weight-drop	TSC	SD	male	Tanshinone ⅡA	12	12	20 mg/kg	NS	IV	4 wks

TSC, Thoracic Spinal Cord; CSC, Cervical Spinal Cord; LSC, Lumbar Spinal Cord; NS, Normal saline; I**P,** Intraperitoneal injection; IV, Intravenous injection;

### 3.3 Study quality

**FIGURE 2:**
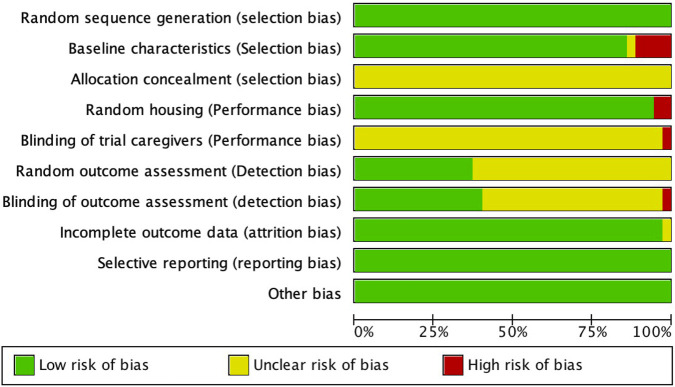
Quality of the included studies.

We independently assessed the risk of bias using the SYCLE tool, which covers six key domains: selection, performance, detection, attrition, reporting, and other biases. Studies meeting the criteria were categorized as low risk, while those not meeting the criteria were considered high risk. Studies with unclear bias risk descriptions were classified as having an unclear risk. Disagreements were resolved through mutual discussion. A detailed assessment of study quality is provided in Figure 2.

### 3.4 Meta-analysis

#### 3.4.1 BBB scale

**FIGURE 3:**
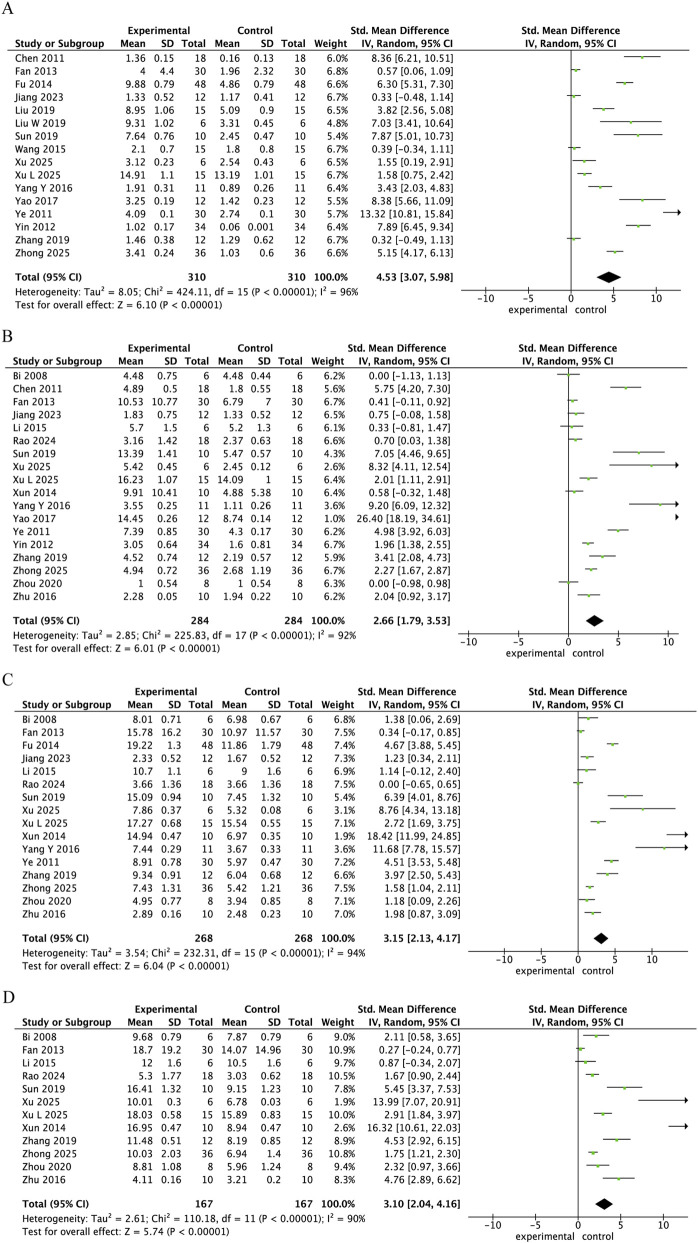
Forest plot comparing BBB scores between the danshen extract treatment group and control group at 3, 7, 14, and 21 days post-injury. **(A)** 3 days post-injury; **(B)** 7 days post-injury; **(C)** 14 days post-injury; **(D)** 21 days post-injury. Each panel displays the pooled standardized mean difference (SMD) and 95% confidence intervals (CI) to evaluate the effect of Danshen extract on motor function recovery at different time points.

Twenty-two studies were included in the analysis of the impact of Danshen extract on motor function in SCI, as measured by the BBB scale. Among these, 16 studies measured outcomes at 3 days post-injury, 18 at 7 days, 16 at 14 days, and 12 at 21 days. The results showed that, in comparison to the control group, the Danshen extract treatment group exhibited significantly improved BBB scores at all time points: 3 days post-injury (SMD = 4.53, 95% CI = 3.07–5.98, p < 0.001), 7 days (SMD = 2.66, 95% CI = 1.79–3.53, p < 0.001), 14 days (SMD = 3.15, 95% CI = 2.13–4.17, p < 0.001), and 21 days (SMD = 3.10, 95% CI = 2.04–4.16, p < 0.001) (Figure 3).

#### 3.4.2 Inflammatory factors in spinal cord tissue

**FIGURE 4:**
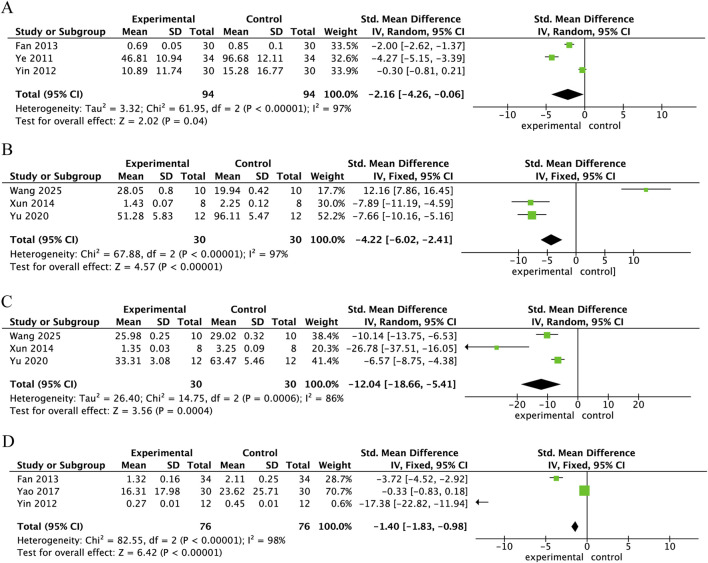
Forest plot comparing inflammatory factors between the danshen extract treatment group and the control group. **(A)** Forest plot of TNF-α expression in spinal cord tissue 24 h post-SCI following Danshen extract treatment; **(B)** Forest plot of TNF-α expression in spinal cord tissue 28 days post-SCI following Danshen extract treatment; **(C)** Forest plot of IL-1β expression in spinal cord tissue 28 days post-SCI following Danshen extract treatment; **(D)** Forest plot of NF-κB expression in spinal cord tissue 24 h post-SCI following Danshen extract treatment.

Three studies reported the levels of TNF-α in spinal cord tissue 24 h after SCI treatment with Danshen extract. The level of TNF-α in the Danshen extract treatment group was markedly reduced compared to the control group (SMD = −2.16, 95% CI: −4.26 to −0.06, p < 0.001), demonstrating a statistically significant difference. Overall heterogeneity was high (I^2^ = 97%, p < 0.001) (Figure 4A). Three studies measured TNF-α levels in spinal cord tissue 28 days following SCI treatment with Danshen extract. The expression of TNF-α in the Danshen extract treatment group was considerably reduced compared to the control group (SMD = −4.22, 95% CI: –6.02 to −2.41, p < 0.001), indicating a statistically significant difference. High overall heterogeneity was observed (I^2^ = 97%, p < 0.001), as illustrated in Figure 4B. Three studies assessed IL-1β levels in spinal cord tissue 28 days following SCI treatment with Danshen extract. IL-1β expression in the Danshen extract treatment group was markedly reduced compared to the control group (SMD = −12.04, 95% CI: –18.66 to −5.41, p < 0.001), indicating a statistically significant difference. The overall heterogeneity was high (I^2^ = 86%, p < 0.001), as displayed in Figure 4C. Three studies measured NF-κB levels in spinal cord tissue 24 h following SCI treatment with Danshen extract. The NF-κB expression was significantly lower in the Danshen extract treatment group compared to the control group (SMD = −1.4, 95% CI: –1.83 to −0.98, p < 0.001), showing a statistically significant difference. High overall heterogeneity was observed (I^2^ = 98%, p < 0.001), as illustrated in Figure 4D.

#### 3.4.3 Apoptosis markers after spinal cord injury

**FIGURE 5:**
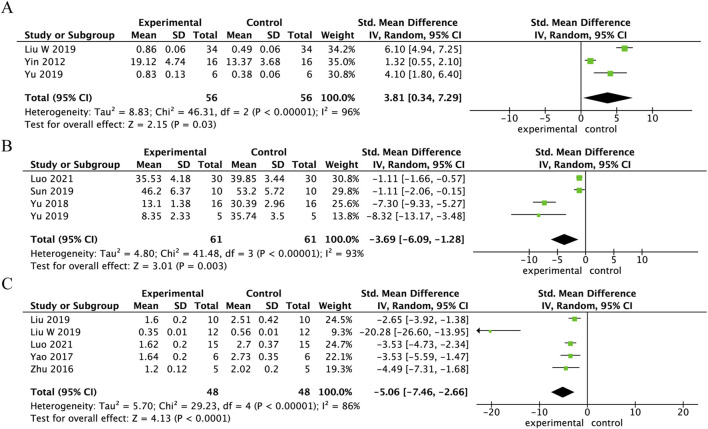
Forest plot comparing apoptosis markers in spinal cord tissue after SCI treatment with Danshen extract. **(A)** Forest plot of BCL-2 expression in spinal cord tissue 3 days post-SCI following Danshen extract treatment; **(B)** Forest plot of TUNEL-positive cells in spinal cord tissue 3 days post-SCI following Danshen extract treatment; **(C)** Forest plot of caspase-3 activity in spinal cord tissue 7 days post-SCI following Danshen extract treatment.

Three studies measured BCL-2 expression in spinal cord tissue 3 days following SCI treatment with Danshen extract. BCL-2 expression was significantly higher in the Danshen extract treatment group compared to the control group (SMD = 3.81, 95% CI: 0.34 to 7.29, p < 0.001), indicating a statistically significant difference. The overall heterogeneity was high (I^2^ = 96%, p = 0.03), as shown in Figure 5A. Three studies documented the count of TUNEL-positive cells in spinal cord tissue 3 days following SCI treatment with Danshen extract. The count of TUNEL-positive cells was significantly reduced in the Danshen extract treatment group compared to the control group (SMD = −3.69, 95% CI: –6.09 to −1.28, p < 0.001), showing a statistically significant difference. High overall heterogeneity was observed (I^2^ = 93%, p = 0.003), as illustrated in Figure 5B. Five studies assessed Caspase-3 activity in spinal cord tissue 7 days following SCI treatment with Danshen extract. Caspase-3 activity in the Danshen extract treatment group was significantly reduced compared to the control group (SMD = −5.06, 95% CI: −7.46 to −2.66, p < 0.001), reflecting a statistically significant difference. High overall heterogeneity was observed (I^2^ = 86%, p < 0.05), as depicted in Figure 5C.

#### 3.4.4 Malondialdehyde (MDA) levels and superoxide dismutase (SOD)

**FIGURE 6:**
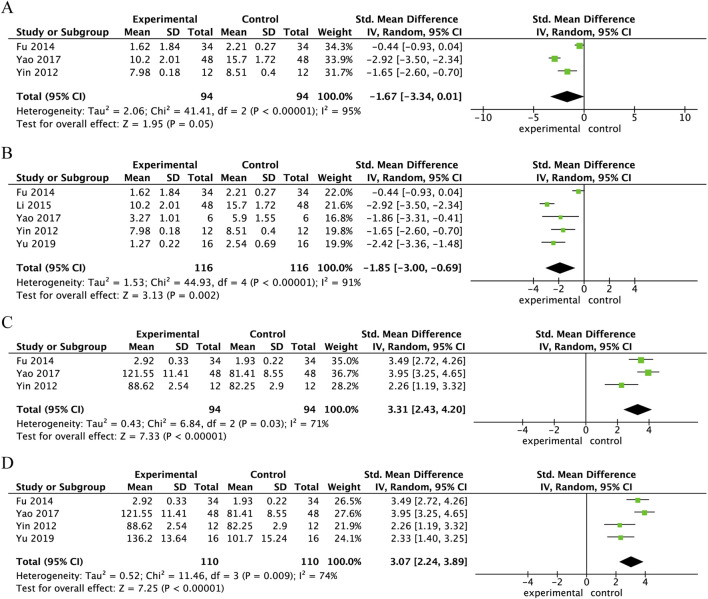
Forest plot comparing MDA levels and SOD activity in spinal cord tissue after SCI treatment with danshen extract. **(A)** Forest plot of MDA levels in spinal cord tissue 24 h post-SCI following Danshen extract treatment; **(B)** Forest plot of MDA levels in spinal cord tissue 3 days post-SCI following Danshen extract treatment; **(C)** Forest plot of SOD activity in spinal cord tissue 24 h Post-SCI following Danshen extract treatment; **(D)** Forest plot of SOD activity in spinal cord tissue 3 days post-SCI following Danshen extract treatment.

Three studies measured MDA levels in spinal cord tissue 24 h following SCI treatment with Danshen extract. The MDA levels were notably reduced in the Danshen extract treatment group compared to the control group (SMD = −1.67, 95% CI: −3.34 to 0.01, p = 0.05), with the difference nearing statistical significance. Overall heterogeneity was high (I^2^ = 95%, p < 0.00001), as shown in Figure 6A. Five studies reported the levels of MDA in spinal cord tissue 3 days after SCI treatment with Danshen extract. The MDA levels in the Danshen extract treatment group were significantly lower than those in the control group (SMD = −1.85, 95% CI: −3.00 to −0.69, p = 0.002), with a statistically significant difference. Overall heterogeneity was high (I^2^ = 91%, p < 0.00001), as shown in Figure 6B. Three studies assessed the levels of SOD in spinal cord tissue 24 h after SCI treatment with Danshen extract. The SOD levels were substantially elevated in the Danshen extract treatment group compared to the control group (SMD = 3.31, 95% CI: 2.47–4.2, p < 0.00001), indicating a statistically significant difference. Overall heterogeneity was moderate (I^2^ = 71%, p = 0.03), as shown in Figure 6C. Four studies reported the levels of SOD in spinal cord tissue 3 days after SCI treatment with Danshen extract. The SOD levels were markedly higher in the Danshen extract treatment group compared to the control group (SMD = 3.07, 95% CI: 2.24–3.89, p < 0.00001), demonstrating a statistically significant difference. The overall heterogeneity was moderate (I^2^ = 74%, p = 0.009), as illustrated in Figure 6D.

#### 3.4.5 Water content percentage in injured spinal cord areas

**FIGURE 7:**
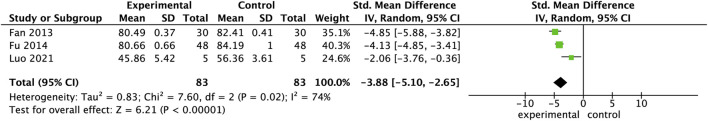
Forest plot comparing water content percentage in injured spinal cord areas following SCI treatment with danshen extract.

Three studies assessed the water content in spinal cord tissue following treatment, providing data on its levels in the context of SCI and the effect of the intervention. Water content in the Danshen extract treatment group was significantly lower than in the control group (SMD = −3.88, 95% CI: −5.10 to −2.65, p < 0.00001), indicating a statistically significant difference. High heterogeneity was observed among the studies (I^2^ = 74%, p = 0.02), though all included studies demonstrated consistent significant differences. The detailed subgroup analysis is shown in Figure 7.

#### 3.4.6 Subgroup analysis

Subgroup analysis revealed that Danshen extract treatment led to a significant improvement in BBB scores at 3 days post-SCI across most subgroups. In terms of different modeling methods, the contusion model (SMD = 3.71, 95% CI: 2.15–5.27, p < 0.01) and the ischemic model (SMD = 5.46, 95% CI: 3.42–7.50, p < 0.01) showed significant improvement, with the ischemic model showing the highest effect. Thoracic SCI (SMD = 3.85, 95% CI: 2.51–5.20, p < 0.01) was more effective than non-thoracic injury (SMD = 3.94, 95% CI: −0.69–8.56, p = 0.10). In terms of species, non-rat models (SMD = 8.36, 95% CI: 6.21–10.51, p < 0.01) showed a significantly higher improvement than rats (SMD = 3.58, 95% CI: 2.32–4.84, p < 0.01). Male animals (SMD = 4.77, 95% CI: 2.63–6.91) showed slightly higher improvement than female animals (SMD = 3.05, 95% CI: 1.42–4.69). Regarding the type of extract, water-soluble phenolic acids (SMD = 5.68, 95% CI: 1.42–9.94) were more effective than lipophilic tanshinones (SMD = 3.95, 95% CI: 2.50–5.40). For dosage, the high-dose group (SMD = 4.01, 95% CI: 1.79–6.23) showed slightly greater improvement than the low-dose group (SMD = 3.81, 95% CI: 2.12–5.50). The detailed subgroup analysis is presented in Table 2.

**Table 2:** Subgroup analysis of BBB score at 3 days after SCI following Danshen treatment

Subgroup	SMD (95% CI)	I²	p value
Model			
Contusion model	3.71 [2.15, 5.27]	95	<0.01
Compression model	4.39 [-3.24, 12.02]	98	0.26
Ischemic model	5.46 [3.42, 7.5]	80	<0.01
Others	0.33 [-0.48, 1.14]	_	0.42
Injury level			
Thoracic spinal cord	3.85 [2.51, 5.2]	95	<0.01
Non-thoracic spinal cord	3.94 [-0.69, 8.56]	98	0.1
Species			
Rat	3.58 [2.32, 4.84]	96	<0.01
Non-rat	8.36 [6.21, 10.51]	_	<0.01
Gender			
Male	4.77 [2.63, 6.91]	97	<0.01
Female	3.05 [1.42, 4.69]	95	<0.01
Compound type			
Lipophilic tanshinones	3.95 [2.5, 5.4]	93	<0.01
Hydrophilic phenolic acids	5.68 [1.42, 9.94]	98	<0.01
Others	0.36 [-0.18, 0.9]	0	0.19
Dose			
≤20 mg/kg	3.81 [2.12, 5.5]	96	<0.01
>20 mg/kg	4.01 [1.79, 6.23]	96	<0.01

Seven days post-SCI, Danshen extract treatment still significantly promoted the recovery of motor function. The contusion model (SMD = 3.30, 95% CI: 2.18–4.42, p < 0.01) showed the most notable improvement, while the compression model (SMD = 3.03, 95% CI: −2.21–8.26, p = 0.26) and ischemic model (SMD = 0.33, 95% CI: −0.81–1.47, p = 0.57) did not show significant differences. Thoracic injury (SMD = 3.16, 95% CI: 2.10–4.21, p < 0.01) was more effective than non-thoracic injury (SMD = 1.03, 95% CI: 0.08–1.99, p = 0.03). Non-rat models (SMD = 5.75, 95% CI: 4.20–7.30, p < 0.01) showed significantly greater improvement compared to rats (SMD = 2.42, 95% CI: 1.57–3.27, p < 0.01). Female animals (SMD = 3.06, 95% CI: 1.94–4.18) showed slightly greater improvement than males (SMD = 2.07, 95% CI: 0.65–3.50). Lipophilic tanshinones (SMD = 3.13, 95% CI: 1.86–4.40) were more effective than water-soluble phenolic acids (SMD = 2.17, 95% CI: 0.80–3.54). Regarding dosage, the high-dose group (SMD = 7.66, 95% CI: 2.89–12.42) showed significantly greater improvement compared to the low-dose group (SMD = 2.15, 95% CI: 1.32–2.99). The detailed results are presented in Table 3.

**Table 3:** Subgroup analysis of BBB score at 7 days after SCI following Danshen treatment.

Subgroup	Standardized mean difference (95% confidence interval)	I²	p value
Model			
Contusion model	3.30 [2.18, 4.42]	92	<0.01
Compression model	3.03 [-2.21, 8.26]	98	0.26
Ischemic model	0.33 [-0.81, 1.47]	98	0.57
Others	0.72 [-0.2, 1.25]	0	<0.01
Injury level			
Thoracic spinal cord	3.16 [2.1, 4.21]	93	<0.01
Non-thoracic spinal cord	1.03 [0.08, 1.99]	71	0.03
Species			
Rat	2.42 [1.57, 3.27]	92	<0.01
Non-rat	5.75 [4.2, 7.3]	_	<0.01
Gender			
Male	2.07 [0.65, 3.5]	94	0.004
Female	3.06 [1.94, 4.18]	91	<0.01
Compound type			
Lipophilic tanshinones	3.13 [1.86, 4.4]	92	<0.01
Hydrophilic phenolic acids	2.17 [0.8, 3.54]	94	0.002
Others	_	_	_
Dose			
≤20 mg/kg	2.15 [1.32, 2.99]	92	<0.01
>20 mg/kg	7.66 [2.89, 12.42]	95	0.002

Fourteen days post-SCI, Danshen extract treatment still demonstrated significant improvements in neurological function. The contusion model (SMD = 3.73, 95% CI: 2.59–4.87, p < 0.01) showed the most significant improvement, while the compression model (SMD = 0.34, 95% CI: −0.17–0.85, p = 0.19), ischemic model (SMD = 2.94, 95% CI: −0.51–6.39, p = 0.10), and other models (SMD = 0.57, 95% CI: −0.62–1.77, p = 0.35) showed no significant differences. Both thoracic injury (SMD = 2.85, 95% CI: 1.85–3.85, p < 0.01) and non-thoracic injury (SMD = 2.89, 95% CI: 0.87–4.91, p = 0.005) showed significant improvement. Male animals (SMD = 3.30, 95% CI: 0.89–5.70) showed slightly greater improvement compared to female animals (SMD = 2.91, 95% CI: 1.89–3.93). In terms of drug type, “other” compounds (SMD = 3.97, 95% CI: 2.50–5.43) had the highest effect, followed by water-soluble phenolic acids (SMD = 2.94, 95% CI: 1.53–4.36) and lipophilic tanshinones (SMD = 2.82, 95% CI: 1.34–4.31). The high-dose group (SMD = 4.12, 95% CI: 3.90–4.35) showed greater improvement than the low-dose group (SMD = 1.90, 95% CI: 1.78–2.02). The detailed results are presented in Table 4.

**Table 4:** Subgroup analysis of BBB score at 14 days after SCI following Danshen treatment.

Subgroup	SMD (95% CI)	I²	p value
Model			
Contusion model	3.73 [2.59, 4.87]	88	<0.01
Compression model	0.34 [-0.17, 0.85]	_	0.19
Ischemic model	2.94 [-0.51, 6.39]	95	0.1
Others	0.57 [-0.62, 1.77]	79	0.35
Injury level			
Thoracic spinal cord	2.85 [1.85, 3.85]	92	<0.01
Non-thoracic spinal cord	2.89 [0.87, 4.91]	92	0.005
Species			
Rat	2.38 [2.27, 2.48]	99	<0.01
Non-rat	_	_	_
Gender			
Male	3.3 [0.89, 5.7]	96	0.007
Female	2.91 [1.89, 3.93]	90	<0.01
Compound type			
Lipophilic tanshinones	2.82 [1.34, 4.31]	92	<0.01
Hydrophilic phenolic acids	2.94 [1.53, 4.36]	94	<0.01
Others	3.97 [2.5, 5.43]	_	<0.01
Dose			
≤20 mg/kg	1.9 [1.78, 2.02]	99	<0.01
>20 mg/kg	4.12 [3.9, 4.35]	98	<0.01

Twenty-one days post-SCI, Danshen extract treatment still demonstrated significant improvements in motor function. The contusion model (SMD = 4.22, 95% CI: 2.82–5.63, p < 0.01) and the ‘other’ models (SMD = 1.67, 95% CI: 0.90–2.44, p < 0.01) showed significant improvement, while the compression model (SMD = 0.27, 95% CI: −0.24–0.77, p = 0.31) and ischemic model (SMD = 0.87, 95% CI: –0.34–2.07, p = 0.16) showed no significant differences. Thoracic injury (SMD = 3.48, 95% CI: 2.23–4.74, p < 0.01) was more effective than non-thoracic injury (SMD = 1.91, 95% CI: −0.09–3.91, p = 0.06). Female animals (SMD = 3.35, 95% CI: 2.25–4.44) showed slightly greater improvement than male animals (SMD = 2.75, 95% CI: 0.45–5.04). In terms of drug type, “other” compounds (SMD = 4.53, 95% CI: 2.92–6.15) had the highest effect, followed by water-soluble phenolic acids (SMD = 3.42, 95% CI: 1.57–5.27) and lipophilic tanshinones (SMD = 2.40, 95% CI: 1.21–3.59). The low-dose group (SMD = 3.22, 95% CI: 2.04–4.39) showed better improvement than the high-dose group (SMD = 2.66, 95% CI: −0.93–6.25). The detailed results are presented in Table 5.

**Table 5:** Subgroup analysis of BBB score at 21 days after SCI following Danshen treatment

Subgroup	SMD (95% CI)	I²	p value
Model			
Contusion model	4.22 [2.82, 5.63]	87	<0.01
Compression model	0.27 [-0.24, 0.77]	_	0.31
Ischemic model	0.87 [-0.34, 2.07]	_	0.16
Others	1.67 [0.9, 2.44]	_	<0.01
Injury level			
Thoracic spinal cord	3.48 [2.23, 4.74]	91	<0.01
Non-thoracic spinal cord	1.91 [-0.09, 3.91]	84	0.06
Species			
Rat	_	_	_
Non-rat	_	_	_
Gender			
Male	2.75 [0.45, 5.04]	92	0.02
Female	3.35 [2.25, 4.44]	83	<0.01
Compound type			
Lipophilic tanshinones	2.4 [1.21, 3.59]	81	<0.01
Hydrophilic phenolic acids	3.42 [1.57, 5.27]	92	<0.01
Others	4.53 [2.92, 6.15]	_	<0.01
Dose			
≤20 mg/kg	3.22 [2.04, 4.39]	91	<0.01
>20 mg/kg	2.66 [-0.93, 6.25]	92	0.15

#### 3.4.7 Sensitivity analysis and publication bias

**FIGURE 8:**
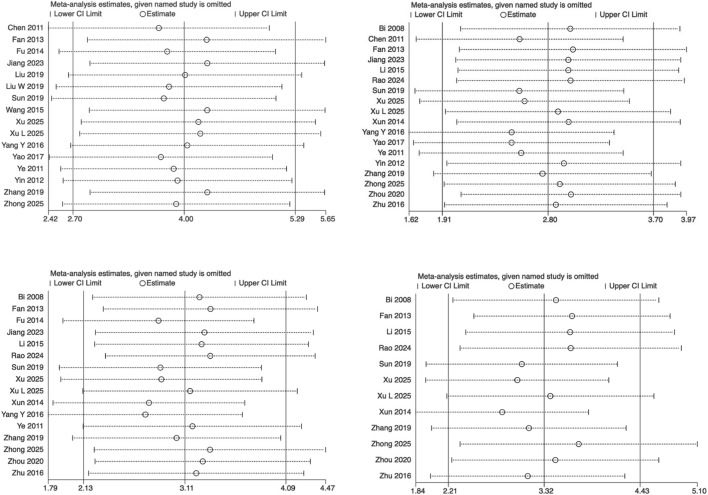
Sensitivity analysis of BBB score outcomes at 3, 7, 14, and 21 days post‐spinal cord injury.

Studies showing no significant changes in heterogeneity indices or 95% confidence intervals were excluded, highlighting minimal variation between studies and reinforcing the reliability of the meta-analysis findings (Figure 8).

## 4 Discussion

This meta-analysis demonstrates that Danshen extract notably enhanced motor function in SCI animal models and mitigated the injury response through various mechanisms. Specifically, Danshen extract substantially lowered the levels of inflammatory markers, including TNF-α and IL-1β, in spinal cord tissue, thereby alleviating the inflammatory response (Figures 4A–C). Furthermore, Danshen extract helped mitigate oxidative stress by decreasing MDA levels and enhancing the activity of SOD, thus supporting the cellular defense mechanisms against oxidative damage (Figures 6A–D). Furthermore, Danshen extract inhibited apoptosis after SCI by upregulating BCL-2 expression, reducing TUNEL-positive cells, and significantly lowering Caspase-3 activity (Figures 5A–C). Crucially, treatment with Danshen extract markedly reduced spinal cord edema and lowered the water content in the injured regions, contributing to the overall protection of spinal cord tissue (Figure 7). These results suggest that Danshen extract, through its anti-inflammatory, antioxidant, anti-apoptotic, and edema-reducing effects, holds potential for SCI treatment and may become an effective neuroprotective agent.

Neurological recovery is a critical indicator of recovery after SCI. The analysis revealed that Danshen extract significantly enhanced the BBB scores at various time points, including 3, 7, 14, and 21 days, indicating that Danshen extract has a sustained promoting effect on motor function after SCI (Figure 3). Consistently, a meta-analysis of curcumin in experimental SCI models (24 studies) reported a significant improvement in functional recovery (BBB/BMS) with a pooled effect size of SMD = 3.38 (95% CI 2.54–4.22, p < 0.001), and daily administration produced stronger effects than single-dose or weekly regimens (Kahuripour et al., 2022). Notably, the most significant treatment effect was observed at 3 days post-injury (Figure 3), suggesting that early intervention may play a crucial role in promoting neurological recovery. The intrinsic regenerative capacity after SCI is widely acknowledged to be limited. However, treatments designed to promote nerve repair and regeneration could potentially recover some spinal cord function (Wu Y-Y. et al., 2025). Following SCI, the restoration of motor function not only indicates the repair of the local spinal cord injury but also serves as a reflection of the rebuilding of neural circuits and the promotion of neuroplasticity, which are crucial for functional recovery (Hu et al., 2023). Improvement in motor function generally indicates the effective progression of biological processes such as neuronal survival, axonal regeneration, and myelin repair (Nagappan et al., 2020). Particularly in the acute phase of SCI, such as within the first 3 days after injury, the injury site experiences heightened levels of inflammation, oxidative stress, and apoptosis, which significantly contribute to tissue damage and impairment. Interventions during this phase can minimize secondary injury and provide a more favorable microenvironment for later neural remodeling and functional recovery (Yin et al., 2012; Zhong et al., 2025). Therefore, early intervention is particularly important in SCI treatment, as it can significantly alleviate injury and promote early neurological recovery.

In the present meta-analysis, Danshen extract significantly improved the BBB scores at multiple time points after SCI (Figure 3), with Danshen extract improving neurological function through multiple mechanisms (Zhang et al., 2019). Across the included studies, the most frequently investigated Danshen constituents in SCI models were tanshinone IIA and salvianolic acid B, both of which have been reported to improve functional recovery and attenuate secondary injury after SCI (Yin et al., 2012; Yao et al., 2017; Zhou et al., 2020). First, Danshen extract significantly reduced inflammatory factors in the spinal cord through its anti-inflammatory effects, thereby mitigating secondary inflammatory damage (Yu and Qian, 2020; Wang et al., 2025). Research has demonstrated that inflammation is a critical factor in the exacerbation of injury in the early stages of SCI. Early intervention to control inflammation has been shown to reduce additional neuronal damage, potentially improving long-term outcomes (Anwar et al., 2016). Second, Danshen extract alleviated oxidative stress by reducing the production of the oxidative stress marker MDA and increasing the activity of the antioxidant enzyme SOD (Figures 6A–D), thereby attenuating cell damage and apoptosis caused by oxidative stress (Yao et al., 2017; Yu et al., 2019). Consistent with our findings, a recent systematic review and meta-analysis of Ginkgo biloba extract in rat SCI models reported improved locomotor recovery (WMD = 2.09, 95% CI 1.59–2.59, p < 0.00001) and reduced oxidative stress/inflammation, including decreased iNOS (WMD = −22.17, 95% CI −35.45 to −8.90, p < 0.00001) and MDA (SMD = −1.43, 95% CI −5.05 to 2.20) (Wu Z-M. et al., 2025). Furthermore, Danshen extract inhibited neuronal apoptosis after SCI by regulating the expression of apoptosis-related proteins (such as Bcl-2 and Caspase-3), further promoting neuronal survival and functional recovery (Wei et al., 2019; Luo et al., 2022). Ultimately, Danshen extract has the potential to decrease the water content in the injured regions of the spinal cord, which could help alleviate swelling and improve tissue preservation (Figure 7). Through its effects on improving microcirculation, antioxidation, anti-inflammation, and promoting neuroprotection and myelin regeneration, Danshen extract significantly reduced edema after SCI and facilitated the repair of the injured areas (Zhang et al., 2016).

Our subgroup analysis found that, in ischemia/reperfusion injury models, the therapeutic effects of Danshen extract were generally more pronounced within a few days after ischemia, particularly during the treatment window around 3 days post-ischemia (Table 2). Immediately following SCI, there is a rapid increase in inflammatory cytokines like TNF, IL-1, and IL-6. This upregulation contributes to cell membrane damage and vascular leakage, initiating a cascade of secondary injury that exacerbates tissue damage and impairs recovery. Cytokines and chemokines are widely upregulated, and immune cell infiltration occurs, with neutrophils peaking around 24 h post-injury and gradually decreasing over the next 7–10 days (Hellenbrand et al., 2021). Hydrophilic phenolic acid compounds are more effective within the first 3 days post-SCI, mainly because they can quickly and effectively act during the peak of the inflammatory and oxidative stress responses. Their high water solubility allows them to rapidly penetrate the blood-brain barrier and reach the injury site (Fan et al., 2013), where they exhibit multiple actions, including anti-inflammatory, antioxidant, and vascular repair properties. In contrast, lipophilic tanshinones, due to their poorer solubility, may not show as significant an effect during this critical window (Jia et al., 2023). Available CNS-related pharmacokinetic evidence based on blood–brain microdialysis after oral administration of Danshen extract suggests that hydrophilic phenolic acids such as danshensu can be detected in brain dialysates, whereas salvianolic acid B may be undetectable under the same conditions, supporting the importance of exposure and formulation when interpreting early therapeutic windows (Zhang et al., 2011). In addition, BBB transport studies indicate that danshensu can cross the BBB with P-gp involvement, whereas tanshinone IIA brain penetration may be restricted by efflux transporters, highlighting that CNS exposure may differ substantially among Danshen constituents (Yu et al., 2011). Future studies should explore the optimal formulations and delivery methods for the active components of Danshen extract to enhance their bioavailability and ensure targeted action. Furthermore, higher doses (>20 mg/kg) ensure that the active components of Danshen extract reach sufficient concentrations in the target tissue, further enhancing its antioxidant, anti-inflammatory, and tissue repair functions. This has a significant advantage in improving local blood flow, reducing the injury area, and promoting functional recovery. Therefore, the use of Danshen extract within 3 days post-ischemia and at higher doses can effectively regulate the repair and regeneration processes, alleviating ischemic damage and accelerating tissue repair. Higher doses are necessary to ensure the active components reach sufficient concentrations in the target tissues, which is critical for therapeutic efficacy. Future clinical trials should consider these dosages and determine whether they are feasible and safe for human use. Importantly, dose selection for future clinical studies should be guided by pharmacokinetic/pharmacodynamic relationships rather than animal dose alone, and standardized reporting of formulation, route of administration, and systemic exposure would strengthen cross-study comparability and clinical dose rationale.

While the meta-analysis conducted in this study offers compelling evidence supporting the therapeutic potential of Danshen extract for SCI, it is important to acknowledge several limitations that may impact the overall conclusions. First, the included studies exhibited significant heterogeneity, including variations in SCI models, species, dosing regimens, and outcome measures, which may affect the comparability and generalizability of the results. Second, some studies had methodological flaws, such as insufficient bias control in random sequence generation and blinding, which may impact the accuracy of the results (Figure 2). Additionally, while animal studies suggest that Danshen extract has neuroprotective effects, the differences between animal models and human physiology require further validation for clinical translation. Many studies reported high standardized mean differences (SMDs). While larger effect sizes may indicate strong therapeutic effects, high SMD values should be interpreted with caution, as possible reasons include small sample sizes, methodological differences, or variations in outcome measures. Finally, the diversity in the active components of Danshen extract, formulation types, and dosages may also influence the treatment outcomes. To build on these findings, future studies should focus on refining experimental methodologies and investigating the clinical applicability of Danshen extract to fully understand its potential as a therapeutic agent.

## 5 Conclusion

This meta-analysis indicates that Danshen extract has significant therapeutic effects in the treatment of SCI, particularly in improving motor function, reducing inflammation, oxidative stress, apoptosis, and edema. Danshen extract notably enhanced BBB scores at 3, 7, 14, and 21 days post-injury. Furthermore, it significantly reduced inflammatory markers like TNF-α and IL-1β, as well as oxidative stress indicators such as MDA, while simultaneously boosting the activity of antioxidant enzymes, including SOD, in the spinal cord. Danshen extract also demonstrated the ability to inhibit apoptosis, decrease the number of TUNEL-positive cells, and significantly reduce spinal cord edema. These findings highlight the therapeutic potential of Danshen extract for SCI treatment. However, to fully validate its clinical applicability, further refinement of study designs and additional clinical research are necessary.

## Data Availability

The original contributions presented in the study are included in the article/supplementary material, further inquiries can be directed to the corresponding authors.
